# Contemporary screen use and symptoms of muscle dysmorphia among a national sample of Canadian adolescents and young adults

**DOI:** 10.1007/s40519-023-01550-7

**Published:** 2023-02-15

**Authors:** Kyle T. Ganson, Laura Hallward, Rachel F. Rodgers, Alexander Testa, Dylan B. Jackson, Jason M. Nagata

**Affiliations:** 1 Factor-Inwentash Faculty of Social Work, University of Toronto, 246 Bloor Street W, Toronto, ON M5S 1V4 Canada; 2 School of Kinesiology, Western University, London, ON Canada; 3 Department of Applied Psychology, Northeastern University, Boston, MA USA; 4 Department of Psychiatric Emergency & Acute Care, Lapeyronie Hospital, Montpellier, France; 5 Department of Management, Policy and Community Health, University of Texas Health Science Center at Houston, Houston, TX USA; 6 Department of Population, Family, and Reproductive Health, Johns Hopkins Bloomberg School of Public Health, Johns Hopkins University, Baltimore, MD USA; 7 Department of Pediatrics, University of California, San Francisco, CA USA

**Keywords:** Screen time, Social media, Muscle dysmorphia, Adolescents, Young adults, Canada

## Abstract

**Purpose:**

Screen time has been previously linked to body dissatisfaction and eating disorder behaviors. However, less is known about whether use of common forms of screen technology is associated with symptoms of muscle dysmorphia (MD), which was the aim of this study.

**Methods:**

Data from the Canadian Study of Adolescent Health Behaviors (*N* = 2538) were analyzed. Associations between hours of use of six contemporary forms of recreational screen time, as well as total screen time, and symptoms of MD were determined using multiple linear regression models, stratified by gender, and adjusting for potential confounders.

**Results:**

Among both men and women, greater total screen time and texting were associated with greater symptoms of MD; however, differences emerged across the screen time modalities by gender. Among women, video chatting was most strongly associated with symptoms of MD, while social media use was most strongly associated with symptoms of MD among men.

**Conclusion:**

Findings add to the growing literature documenting the potentially harmful correlates of screen time by including MD symptomatology. Findings have important implications for health care, public health, and policymaking professionals.

**Level of evidence:**

Level V, cross-sectional descriptive study.

**Supplementary Information:**

The online version contains supplementary material available at 10.1007/s40519-023-01550-7.

## Introduction

The use of screens and social media is high among adolescents and young adults [1, 2], and time spent on screens has significantly increased during the COVID-19 pandemic [3]. Many adolescents and young adults use screens and social media as tools for social connection; however, screen time and social media use may create adverse psychological, emotional, and social impacts [4, 5]. Research has documented that greater time spent on screens and social media was associated with depression, anxiety, physical inactivity, higher body mass index (BMI), poor body image, and eating disorders [5–9].

A robust body of research has documented strong associations between screen time, social media use, and poor body image, which can lead to behaviors aimed at changing weight and/or appearance [9–12]. These relationships align with theoretical models of eating disorders. For example, the Tripartite Influence Model describes how parents, peers, and media—including social media—can precipitate the development of body dissatisfaction and traditional eating disorder behaviors (i.e., restricting, binging, purging) due to the internalization of body ideals and social pressures [13, 14]. This same model has also been used to examine the development of muscle dissatisfaction and muscularity-oriented behaviors, particularly among boys and men [15–17], and in relation to screen time and social media use [12, 18].

The effects of social media use on body image and eating disorder behaviors can be exacerbated by the algorithms used by social media companies, as highlighted in recent high profile media stories [19, 20]. These algorithms create “rabbit holes”, whereby individual users see more of the content that they engage with, either actively via following specific accounts or “liking” content, or passively via spending longer time on photos/videos. This may ultimately perpetuate the unrealistic body ideals, which are commonly posted on social media [21–23], and precipitate attempts to change one’s body [8]. Alternatively, prior research has documented the negative body image effects of taking and posting “selfies” on social media [24, 25], as well as appearance concerns related to use of video chatting platforms (i.e., Zoom) [26, 27].

Along with the potential negative effects of social media use previously outlined, greater time on screens may displace physical activity given the inactive nature of many forms of screen time (i.e., watching TV or videos) [28]. Lower physical inactivity may also increase body dissatisfaction due to potential weight gain or mindless eating that can occur during this time [6, 29]. Additionally, it has been posited that engagement with screens and social media, such as watching videos on YouTube or following influencers on social media, may be mechanisms to learn about specific exercise and eating regimens to strive for specific gendered body ideals [30].

To date, however, much of the research on screen time and social media use has focused on efforts to lose weight, such as dieting [9, 11, 31]. This has left a significant gap in our knowledge on the associations between specific screen time and social media use modalities and muscularity-oriented body concerns and behaviors. One recent study showed that screen time and social media use were not associated with, or protective of, muscle-building behaviors (i.e., changing eating, exercising more, or use of protein powders all in an attempt to increase muscle mass) among both men and women [32]. Conversely, an additional study among adolescent boys and girls found relationships between greater social media use and higher muscular ideal internalization and muscle-building behaviors [33].

However, more research is needed to fully understand the potential associations between screen time and social media use and symptoms of muscle dysmorphia (MD), which is characterized as the pathological pursuit of muscularity [34, 35]. MD is largely understudied in large community samples, including understanding social correlates of MD symptoms, and is more common among men than women [36]. Given the significant research attention on the relationships between screen time and social media use and thinness-oriented body dissatisfaction and eating disorder behaviors, similar investigations are warranted for MD. Therefore, the aim of the current study was to determine the associations between screen time and symptoms of MD among a large, national sample of Canadian adolescents and young adults, stratified by gender. Given that there are gender differences in screen use (i.e., males generally reporting more screen time compared to females) [1, 3] and males predominately experience MD symptoms compared to females [36–38] investigating the associations between screen time and symptoms of MD by gender provides greater understanding of these associations which can be tailored to prevention and intervention efforts. Based on prior theory (i.e., Tripartite Influence Model) and research previously described, it was hypothesized that there would be significant associations between greater screen time and greater symptoms of MD.

## Methods

Data from adolescent and young adult participants (*N* = 2538) from the Canadian Study of Adolescent Health Behaviors were analyzed. The convenience sample was recruited via Instagram and Snapchat advertisements, without targeting specific populations, from November to December 2021. Eligibility criteria included: (a) living in Canada, (b) ages 16–30 years old, and (c) able to understand English. Participants completed the survey online via Qualtrics and were offered the opportunity to enter a draw to win one of two Apple iPads or one of 20 $25 Starbucks gift cards as compensation for completing the survey. Ethical approval for the study (#41707) was received at the University of Toronto and informed consent was obtained from all participants.

## Measures

### Dependent variable: muscle dysmorphia symptoms

Symptoms of MD were assessed with the 13-item Muscle Dysmorphic Disorder Inventory (MDDI; [39]). Participants responded on a Likert-scale from 1 (never) to 5 (always) for each item, and a total score was calculated by summing all items [39]. The MDDI is among the most widely used measures of MD symptoms [40]. Internal reliability for the present study using Cronbach’s alpha was acceptable for women (*α* = 0.73) and good for men (*α* = 0.80).

### Independent variables: screen time

Participants were asked to indicate how much time per day they spend on a screen in a typical week (Monday through Sunday) for recreational purposes (i.e., not time included for school or work). Screen time included watching TV shows or movies; watching videos (such as YouTube); playing video games on a computer, console, phone, or other device; texting on a cell phone, tablet, or computer; visiting social networking sites (i.e., Facebook, Twitter, Instagram); and video chatting (i.e., Skype, Facetime). For each type of screen time, participants could indicate 0 h, 0.25 h, 0.5 h, 0.75 h, or anywhere from 1 to 12 h or more (top coded at 12 h or more). Total screen time was also calculated by summing all screen time items. Each screen time item was coded as a continuous variable. These screen time items and coding are derived from prior research [1, 3, 7].

### Sociodemographic characteristics

The sociodemographic variables measured included age, body mass index (BMI; kilogram/meters^2^), race/ethnicity, gender identity, sexual identity, and the highest level of education completed. These variables were adjusted for based on prior research [32, 38]. For the purposes of this study, only cisgender people were included (referred to as women and men) due to small cell sizes for transgender and gender non-binary identifying participants.

### Statistical analysis

All analyses were conducted using Stata 17 [41]. Descriptive statistics using means (M) and standard deviations (SD), and frequencies (percentages) were used to describe the characteristics of the sample. Independent samples *t* test and chi-square tests were used to determine differences between genders for continuous variables and categorial variables, respectively. Multiple linear regression models were estimated to determine the associations between the independent variables (all screen time variables modeled separately) and the dependent variable (MDDI total score), stratified by gender (women, men), adjusting for the sociodemographic variables. Assumptions of linear regression were checked, including linearity (residual plot, augmented component-plus-residual plot), homoscedasticity (Cook-Weisberg test for heteroscedasticity), normality (kernel density plot, standardized normal probability plot), and absence of multicollinearity (variance inflation factors). Statistical significance for all analyses was defined as two-sided *p* < 0.05. Listwise deletion was used to account for missing data, which is robust to the missing at random assumptions (as in the current study Little’s MCAR test *p* > 0.05) and raises minimal issues with statistical power given the sample size of the study [42, 43]. Participants were considered in the final analyses if they had valid responses to all variables included. See Supplemental Table 1 for an outline of missing data per variable.

## Results

The sample was comprised of 58.2% women. Men reported significantly greater time watching videos, playing video games, and total screen time, while women reported significantly greater time on social media. Men had significantly greater MD symptoms compared women (Table 1).

**Table 1:** Characteristics of 2538 participants from the Canadian Study of Adolescent Health Behaviors

	Women *n* = 1477	Men *n* = 1061	*p*^a^
	*n* (%)	*n* (%)	
Age [M (SD)]	23.1 (3.9)	22.8 (3.9)	0.108
BMI [M (SD)]	24.3 (5.7)	25.0 (3.9)	0.002
Race/ethnicity			< 0.001
White	963 (62.0)	610 (57.5)	
Black	48 (3.3)	33 (3.1)	
Latino	28 (1.9)	34 (3.2)	
East Asian	137 (9.3)	115 (10.8)	
South Asian	78 (5.3)	108 (10.2)	
Middle Eastern	28 (1.9)	34 (3.2)	
Indigenous	22 (1.5)	11 (1.0)	
Other	19 (1.3)	17 (1.6)	
Multi-racial	154 (10.4)	99 (9.3)	
Sexual identity			< 0.001
Heterosexual	846 (57.3)	774 (70.2)	
Gay/lesbian	36 (2.4)	153 (14.4)	
Bisexual	351 (23.8)	92 (8.7)	
Queer, questioning, or other	244 (16.5)	71 (6.7)	
Education			0.134
High school diploma or less	615 (41.7)	478 (45.1)	
College or undergraduate degree	646 (43.8)	458 (43.2)	
Master’s degree or higher	190 (12.9)	114 (10.7)	
Other	24 (1.6)	11 (1.0)	
Screen time [M (SD)]			
Watching TV	1.9 (1.8)	1.8 (2.0)	0.112
Watching videos	1.2 (1.5)	1.7 (1.6)	< 0.001
Video games	0.4 (1.0)	1.1 (1.4)	< 0.001
Social media	2.3 (1.8)	1.9 (1.8)	< 0.001
Texting	1.7 (2.0)	1.6 (1.9)	0.378
Video chat	0.5 (1.1)	0.5 (1.2)	0.814
Total screen time	15.4 (10.9)	16.3 (10.6)	0.040
MDDI total score [M (SD)]	30.2 (7.3)	33.9 (8.6)	< 0.001

BMI, body mass index; M, mean; SD, standard deviation; MDDI, Muscle Dysmorphic Disorder Inventory

^a^*p* values determined using independent samples *t* test for continuous variables and chi-square tests for categorial variables

Results from multiple linear regression analyses revealed several significant associations between screen time and symptoms of MD (Table 2). Among women, greater time watching TV and videos, texting, video chatting, and total screen time were significantly associated with greater symptoms of MD. Among men, greater time on social media, texting, and total screen time were significantly associated with greater symptoms of MD. See Supplemental Table 2 for results among the overall sample.

**Table 2:** Associations between screen time and MDDI total score by gender among participants from the Canadian Study of Adolescent Health Behaviors (*N* = 2538)

	Women *n* = 1477			Men *n* = 1061		
	*n*^a^	*b* (95% CI)^b^	*p*	*n*^a^	*b* (95% CI)^b^	*p*
Watch TV	1181	**0.24 (0.01, 0.48)**	**0.040**	870	0.02 (− 0.26, 0.30)	0.880
Watch videos	1170	**0.35 (0.06, 0.64)**	**0.018**	855	0.32 (− 0.05, 0.68)	0.088
Video games	1181	0.17 (− 0.26, 0.59)	0.444	867	− 0.28 (− 0.70, 0.14)	0.196
Social media	1182	0.20 (− 0.03, 0.43)	0.087	868	**0.99 (0.67, 1.31)**	** < 0.001**
Texting	1181	**0.26 (0.04, 0.47)**	**0.019**	869	**0.66 (0.36, 0.95)**	** < 0.001**
Video chat	1181	**0.42 (0.03, 0.81)**	**0.035**	866	− 0.06 (− 0.52, 0.40)	0.807
Total screen time	1153	**0.18 (0.14, 0.22)**	** < 0.001**	840	**0.22 (0.17, 0.27)**	** < 0.001**

Each cell represented the abbreviated outputs of 14 linear regression models with screen time and social media as the independent variables and MDDI total score as the dependent variable. Values in bold are significant with *p* < 0.05

MDDI, muscle dysmorphic disorder inventory; CI, confidence interval

^a^Number of observations for each individual linear regression model

^b^Analyses adjusted for body mass index, race/ethnicity, sexual identity, and highest level of education completed

## Discussion

The findings from this study are the first known to document associations between several specific screen time modalities and symptoms of MD. Notably, among both women and men, greater total recreational screen time was associated with greater symptoms of MD. Similarly, among both women and men, greater time spent texting was associated with greater symptoms of MD. However, there were several divergent findings between women and men. Among women, greater time spent watching TV and videos and video chatting were associated with greater symptoms of MD, while among men, greater time spent on social media was associated with greater symptoms of MD. These findings contradict recent research showing no relationships between screen time and social media use and muscularity-oriented behaviors [32], however, align with findings among adolescents [33].

While this study did not investigate specific mechanisms or purpose of screen time in relation to symptoms of MD, there are several possible explanations for the findings. First, as posited by the Tripartite Influence Model, it is possible the peer and social influences on screens and social media increase muscle dissatisfaction, resulting in drive for muscularity. For example, cyberbullying commonly occurs via text messages [44], and research has shown connections between bullying and MD [45]. Additionally, the presence of online “influencers”, high profile social media actors with significant influence on their social media following [46], may drive MD-related symptoms and behaviors. It is plausible, for example, that women are watching influencer videos on YouTube that describe engagement in specific regimens aimed at achieving muscular, lean, and toned bodies. This may also be the case for social media use for men.

Second, it is highly possible that body and muscle dissatisfaction increase as time on screens and social media also increases. This may be due to exposure to body ideals or comparisons with body ideals regularly seen on social media, TV, and videos [23, 47]. Additionally, time spent video chatting may increase appearance concerns, particularly given the mirroring effects of seeing oneself on screen and the “touch up” features provided by video chatting applications [26, 27]. Body and muscle dissatisfaction may also arise given the physically inactive nature of screen time and social media use [29].

Lastly, it is possible that time spent on screens and social media are mechanisms to engage in the muscularity-oriented community. For example, use of certain social media platforms may be opportunities for education on exercise and diet regimens and muscle-building supplements and drugs (i.e., whey protein, creatine, anabolic steroids) for users [30]. There is also the possibility of influencing behaviors, particularly among adolescent and young adults who may lack the developmental maturity to think critically about the content they are viewing [48].

Before outlining implications from the findings from this study, several limitations should be noted. First, the sample was collected via a non-probability, convenience sampling method, which may have increased selection bias and may reduce external validity of the findings. However, participants were located in all 13 provinces and territories in Canada. Future research should consider using a nationally representative sample, as well as a longitudinal cohort to identify prospective relationships between screen time and social media use and symptoms of MD. Additionally, future research may consider exploring these associations among individuals over 30 years of age and determining whether there are differences between age groups (i.e., adolescents vs adults). Second, this study did not ask about the specific purposes or content of screen time and social media use. Therefore, explanations used to explain the findings are hypothetical, and additional research is needed to explore these links. Additionally, it is possible that the coding for the screen time measures resulted in underestimates given top coding at 12 or more hours. However, less than 1% of respondents reported 12 or more hours on each of the screen time items. Lastly, unmeasured confounders may have potentially influenced the findings. Strengths the study include the large, diverse, and national sample of adolescents and young adults across all 13 provinces and territories in Canada.

With these limitations and strengths in mind, several important implications can be drawn from the study findings. First, health care and mental health professionals should be aware of the link between screen time and social media use and symptoms of MD. Assessment of screen time and social media use among clients, including the types of technology used, may aid in understanding the connections with symptoms of MD. Similarly, guidance on positive and healthy engagement on screens and social media, including how to limit time spent using these platforms, as well as healthy engagement with peers and content, may curtail the development of symptoms of MD. Finally, health care professionals can provide information and guidance on what is a realistic and appropriate level of muscle mass on a patient-by-patient basis. Public health professionals should incorporate content related to MD into their prevention and intervention efforts focused on screen time and social media use among adolescent and young adults. Additionally, educational efforts should emphasize the high level of unrealistic body ideals found online. Policymakers and technology companies should consider implementing new regulations and terms of use, such as banning specific content or ensuring safety and validity of content shared, to protect the health and well-being of adolescents and young adults.

## Conclusion

The findings from this study describe the relationships between screen time and symptoms of MD among men and women in a large, national sample of adolescents and young adults in Canada. Among both men and women, greater overall screen time was associated with symptoms of MD, while time spent video chatting among women and on social media among men, were most strongly associated with symptoms of MD. Findings underscore the need for more research, including the sociodemographic factors associated with MD symptomatology [49], particularly in Canada, and the incorporation of MD into prevention and intervention efforts targeted at body image and eating disorders in relation to the use of screens.

## What is already known on this subject?

Screen time and social media use have been shown to be associated with body dissatisfaction and thinness-oriented eating disorders and related behaviors. However, little research has investigated whether screen time is connected with muscle dysmorphia, which is characterized as the pathological pursuit of muscularity.

## What this study adds?

Findings delineate the contemporary forms of screen time that are associated with symptoms of muscle dysmorphia among adolescent and young adults. Specifically, among both men and women, greater time texting and total screen time were associated with greater symptoms of muscle dysmorphia. Among women, greater time watching TV, watching videos, and video chatting were also associated with greater symptoms of muscle dysmorphia, while among men, greater time on social media was also associated with greater symptoms of muscle dysmorphia. Findings add to the growing literature that underscores the potentially harmful correlates of excess screen time among adolescents and young adults.

## Supplementary Information

Below is the link to the electronic supplementary material.

Supplementary file1 (DOCX 16 KB)

## Data Availability

Data may be made available upon reasonable request.
