# Supplementary material for: Contemporary screen use and symptoms of muscle dysmorphia among a national sample of Canadian adolescents and young adults
Source: Eat Weight Disord. 2023 Feb 15;28(1):10. doi: 10.1007/s40519-023-01550-7 (PMC9930713; doi:10.1007/s40519-023-01550-7)
Supplement: Supplementary file 1 — Supplementary file1 (DOCX 16 KB) [file 40519_2023_1550_MOESM1_ESM.docx]

| Supplemental Table 1.  Number Missing for Each Variable Included | |
| --- | --- |
|  | Missing  n/2,538 |
| BMI | 437/2,538 |
| Gender | 0/2,538 |
| Race/Ethnicity | 0/2,538 |
| Sexual Identity | 1/2,538 |
| highest level of education completed | 2/2,538 |
| MDDI Total Score | 46/2,538 |
| Watch TV | 22/2,538 |
| Watch Videos | 51/2,538 |
| Video Games | 28/2,538 |
| Social Media | 25/2,538 |
| Texting | 28/2,538 |
| Video Chat | 30/2,538 |
| Total Screen Time | 94/2,538 |
| BMI = Body mass index; MDDI = Muscle Dysmorphic Disorder Inventory | |

| Supplemental Table 2.  Associations between Screen Time and MDDI Total Score among Participants from the Canadian Study of Adolescent Health Behaviors (N = 2,538) | | | |
| --- | --- | --- | --- |
|  | n^a^ | *b* (95% CI)^b^ | p |
| Watch TV | 2,051 | 0.15 (-0.03, 0.33) | 0.099 |
| Watch Videos | 2,025 | **0.34 (0.11, 0.57)** | **0.004** |
| Video Games | 2,048 | -0.10 (-0.39, 0.20) | 0.516 |
| Social Media | 5,050 | **0.54 (0.35, 0.73)** | **< 0.001** |
| Texting | 2,050 | **0.43 (0.25, 0.60)** | **< 0.001** |
| Video Chat | 2,047 | 0.19 (-0.11, 0.49) | 0.207 |
| Total Screen Time | 1,993 | **0.20 (0.17, 0.23)** | **< 0.001** |
| Note. Each cell represented the abbreviated outputs of 7 linear regression models with screen time and social media as the independent variables and MDDI total score as the dependent variable. Values in **bold** are significant with p < 0.05.  ^a^ Number of observations for each individual linear regression model.  ^b^Analyses adjusted for body mass index, gender, race/ethnicity, sexual identity, and highest level of education completed.  MDDI = Muscle Dysmorphic Disorder Inventory; CI = Confidence interval | | | |
